# Whole-mount immunofluorescence staining of blood and lymphatic vessels in murine small intestine

**DOI:** 10.1016/j.xpro.2023.102310

**Published:** 2023-05-13

**Authors:** Satu Paavonsalo, Yelin Subashi, Madeleine H. Lackman, Sinem Karaman

**Affiliations:** 1 Individualized Drug Therapy Research Program, Faculty of Medicine, University of Helsinki, Helsinki 00014, Finland; 2 Wihuri Research Institute, Helsinki 00290, Finland

**Keywords:** Microscopy, Model Organisms

## Abstract

The small intestine is an excellent model for studying changes in vasculature in response to different diseases or gene deletions. Here, we present a protocol for whole-mount immunofluorescence staining of blood and lymphatic vessels in the adult mouse small intestine. We describe the steps for perfusion fixation, tissue sample preparation, immunofluorescence staining, and whole-mount preparation of stained samples. Our protocol will enable researchers to visualize and analyze the intricate network of vessels in the small intestine.

For complete details on the use and execution of this protocol, please refer to Karaman et al. (2022).^1^

## Highlights

• Whole-mount immunofluorescence staining of the adult murine intestine
• Imaging of murine small intestine vasculature with confocal microscopy
• Preparation of silicone-coated plates for whole-mount preparations
• Can be adapted to staining and imaging other cell types or other tissues

## Abstract (editor highlights)

Publisher’s note: Undertaking any experimental protocol requires adherence to local institutional guidelines for laboratory safety and ethics.

## Graphical abstract

**Figure:**
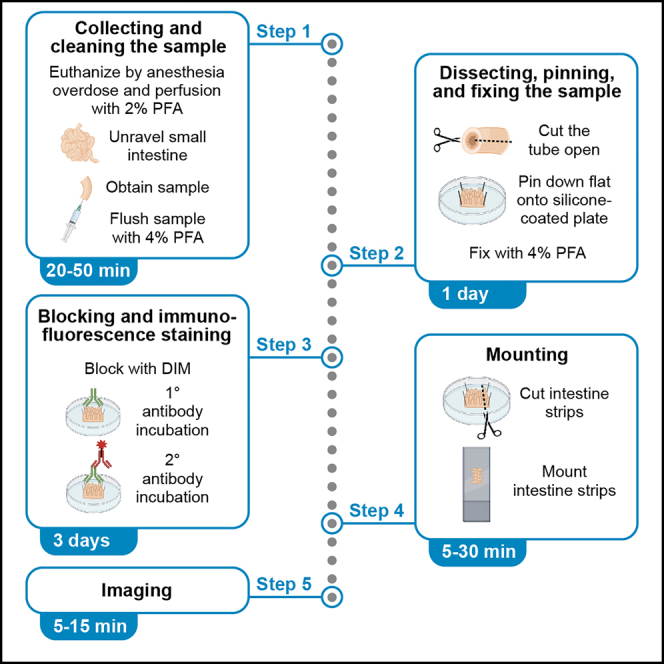

## Before you begin

While this protocol focuses on the staining of blood and lymphatic vessels in the small intestine of adult mice, it can be adapted for other applications. With appropriate modifications to sample preparation, antibody combinations, and mounting procedures, we have successfully used this protocol for endothelial and cell type-specific immunostainings in murine trachea and ear skin. The detailed steps provided below serve as a starting point for customizing the protocol to suit other tissues and cell types of interest.

### Institutional permissions

Animal experiments described in this protocol were approved by the Committee for Animal Experiments of the District of Southern Finland. All animal experiments were conducted in compliance with relevant institutional and national guidelines and regulations. Experimenters must obtain necessary ethical approvals from their institutional or national animal ethics committees before commencing any animal work.

### Prepare buffers

**Timing: 1 h**

1. Prepare buffers as described in materials and equipment.
  a. 1× Phosphate Buffered Saline (PBS).
  b. 2× PBS.
  c. 0.3% Triton X-100 in PBS (0.3% PBST) washing buffer.
  d. 5% Sodium azide in PBS.
  e. Donkey immunomix (DIM).

### Prepare fixing solutions (4%, 2%, and 1% paraformaldehyde in PBS)

**Timing: 1 day**

***Note:*** Approximately 40 mL of 4% paraformaldehyde (PFA) in 1× PBS is needed for this protocol. Below, we describe how to prepare 500 mL of 4% PFA in PBS.

**CRITICAL:** Operate in a chemical fume hood and wear gloves to minimize the possible risks of working with PFA, which is a moderately toxic substance and a carcinogen.

2. Measure 250 mL of Milli-Q (MQ) water in a glass beaker.
3. Heat the water to 60°C using a heating plate with magnetic stirring and cover the beaker with aluminum foil to reduce evaporation. Maintain the liquid temperature at around 60°C.

**CRITICAL:** Measure the temperature of the water regularly and make sure that it does not exceed 65°C to reduce the risk of PFA decomposition.

4. Slowly add 20 g of PFA powder to the 60°C water and increase the stirring speed to mix vigorously.
5. Add one drop (approximately 20 μL) of 10 M NaOH using a glass Pasteur pipette.
6. Once the solution is clear, turn off the heating and add an equal volume (250 mL) of 2× PBS cooled to 4°C.

***Note:*** Using 2× PBS at 4°C will help the solution to reach 20°C–25°C faster, which is required for the upcoming pH measurement.

7. Once the solution reaches 20°C–25°C, measure the initial pH of the solution.

**CRITICAL:** The solution should be at 20°C–25°C for precise pH measurement.

8. Adjust the pH of the solution to 7.4.
  a. Use 6 M HCl solution if the initial pH is higher than 7.4.
  b. Use 10 M NaOH solution if the initial pH is lower than 7.4.

***Note:*** HCl and NaOH solutions should be added dropwise carefully using a glass Pasteur pipette since even small volumes of these solutions can dramatically affect the pH of the solution.

9. Filter the solution into a glass bottle using Whatman filter paper and a glass funnel.
10. Prepare 25 mL of 2% PFA and 5 mL of 1% PFA by diluting the 4% PFA in 1× PBS.
11. Store the PFA solutions protected from light at 4°C for up to 1 week or at −20°C for up to 1 year.

### Prepare the silicone-coated plate

**Timing: 2 days**

**CRITICAL:** Prepare and dry the silicone-coated plates in a fume hood.

12. Thoroughly mix silicone elastomer base with silicone elastomer curing agent manually in a 10 to 1 ratio in a disposable container.
13. Immediately after mixing, pour the mix into molds of interest, such as 6-well cell culture plates or 10 cm Petri dishes as at least 5 mm thick layers.
14. Allow the silicone elastomer to cure for at least 48 h at 20°C–25°C in a fume hood.

**CRITICAL:** Make sure to cure the silicone-coated plates on a flat surface.

***Optional:*** Add insect pins to the plate after the silicone has solidified.

***Note:*** The silicone-coated plate can be reused several times.

### Prepare Mowiol mounting medium

**Timing: 3–4 h**

***Note:*** Less than 1 mL of Mowiol mounting medium is needed for this protocol. Below, we describe how to prepare approximately 25 mL of Mowiol mounting medium.

15. Measure 5 mL of glycerol and add it into a 50–100 mL glass beaker.
16. Add 2.4 g of Mowiol.
17. Add 6 mL of MQ water and mix thoroughly using a heating plate with magnetic stirring. Continue stirring for 2 h at 20°C–25°C.
18. Add 12 mL of 0.2 M Tris (pH 8.5) and heat up to approximately 53°C. Continue stirring until Mowiol has mostly dissolved.
19. Transfer the mixture into a 50 mL tube and centrifuge at 1700–2700 g for 20 min at 20°C–25°C.
20. Divide the supernatant into fresh tubes and store at 20°C–25°C for active use or at −20°C for long-term storage.

## Key resources table

**Table:** 

REAGENT or RESOURCE	SOURCE	IDENTIFIER
**Antibodies**		

Goat polyclonal anti-Mouse Podocalyxin (1:400)	R&D Systems	Cat# AF1556
Rabbit polyclonal to LYVE1 (1:600)	AngioBio	Cat# 11-034
Donkey Anti-Goat IgG (H+L) Highly Cross-Adsorbed Secondary Antibody, Alexa Fluor™ Plus 488 (1:1000)	Invitrogen	Cat# A32814
Donkey Anti-Rabbit IgG (H+L) Highly Cross-Adsorbed Secondary Antibody, Alexa Fluor™ Plus 594 (1:1000)	Invitrogen	Cat# A32754

**Chemicals, peptides, and recombinant proteins**		

Paraformaldehyde (reagent grade, crystalline)	Sigma-Aldrich	Cat# P6148-1 KG
Triton X-100	Fisher BioReagents	Cat# BP151-500
Bovine serum albumin (BSA) lyophilized pH ∼7	Biowest	Cat# P6154-100GR
VECTASHIELD® Antifade Mounting Medium	Vector Laboratories	Cat# H-1000-10
Rompun® vet 20 mg/mL (xylazine)	Bayer	Cat# 6047
Ketaminol® vet 50 mg/mL (ketamine)	Intervet International	Cat# 511485
Clear nail polish	Essence	N/A
SYLGARD™ 184 Silicone Elastomer Kit	Dow	Cat# 1673921
Mowiol® 4–88, Polyvinylalcohol 4–88	Sigma-Aldrich	Cat# 81381-250G
Phosphate buffered saline (PBS) tablets	Medicago	Cat# 09-9400-100
50% Sodium hydroxide (NaOH)	Sigma-Aldrich	Cat# 415413-500ML
37% Hydrochloric acid (HCl)	Acros Organics	Cat# 124630025
Normal donkey serum (NDS)	Biowest	Cat# S2170-500
Sodium azide (NaN_3_)	Sigma-Aldrich	Cat# S2002-100G
Glycerol	Fisher BioReagents	Cat# BP229-1
Trizma® base	Sigma-Aldrich	Cat# T1503-5KG

**Experimental models: Organisms/strains**		

C57BL/6J wild-type mice, sex: male or female, age: 8–12 weeks	The Jackson Laboratory	Strain code: 000664

**Other**		

Disposable animal feeding needles, 20G × 1.5″, 1.9 mm, Flex PTFE (plastic gavage needles)	Cadence Science	REF# 9920
Vannas-Tübingen Spring Scissors (spring scissors)	Fine Science Tools	Cat# 15003-08
BD Micro-Fine Syringes DEMI U100 0.3 mL 30G × 8 mm	Becton Dickinson International	Cat# 324826
Ruler	N/A	N/A
Leica Stereozoom S9D Stereomicroscope	Leica Microsystems	Cat# 10450815
Peristaltic pump LLG-uniPERISTALTICPUMP 1	LLG Labware	Cat# 00010911210035
Metzenbaum Baby Scissors Straight 13 cm (scissors)	Fine Science Tools	Item No. 14018-13
Dumont #2 - Laminectomy Forceps (blunt forceps)	Fine Science Tools	Item No. 11223-20
Dumont #7 Curved forceps (curved forceps)	Fine Science Tools	Item No. 11297-00
Fine scissors, sharp (fine scissors)	Fine Science Tools	Item No. 14060-09
Dumont #5SF Forceps (fine forceps)	Fine Science Tools	Item No. 11252-00
Epredia™ SuperFrost Plus™ Adhesion Microscope slides (25 × 75 × 1 mm)	Epredia	Ref# J1800AMNZ
Microscope cover slips (24 × 40 × 0.16–0.19 mm) (cover slips)	Fisher Scientific	Item no. 11901998
BD Vacutainer® Safety-Lok Wing Needle (infusion needle)	Becton Dickinson International	Cat# 368383
6-well plate	Greiner Bio-one	Cat# 657160
Minutens stainless steel, length 12 mm, diameter 0.20 mm (insect pins)	Ento Sphinx	Ref# 03.20
Stuart SSL3 Gyratory Rocker (Rotator)	Stuart	Cat# 51900-28
Parafilm PM-992 (2 In. X 250 Ft.)	Bemis	Cat# PM-992
Aluminum foil	N/A	N/A
Whatman filter paper	Whatman	Cat# 989410110
12 MP Stand-alone Microscope Camera Flexacam C1	Leica Microsystems	Cat# 12730522
Centrifuge 5430 R	Eppendorf	Cat# 5430R
Vortex-Genie 2	Scientific Industries, Inc.	Cat# SI-0236
Isotemp™ Hot Plate Stirrer	Fisher Brand	Cat#SP88850205

## Materials and equipment

• 1× PBS: add 1 tablet of 1× PBS in 1000 mL of MQ water.

Keep at 20°C–25°C for a month.

• 2× PBS: add 1 tablet of 1× PBS in 500 mL of MQ water.

Keep at 4°C for up to 6 months.

• 0.3% PBST (v/v) (Washing buffer): add 1.5 mL of Triton-X 100 in 498.5 mL of 1× PBS.

Keep at 20°C–25°C indefinitely.

• 5% Sodium azide (w/v): add 25 g of sodium azide in 500 mL of 1× PBS.

Keep at 20°C–25°C indefinitely.

**CRITICAL:** Always operate in a chemical fume hood and wear gloves to minimize the possible risks of working with sodium azide, which is a toxic substance that changes to a gaseous state after mixing with water.

**Table:** DIM (Blocking buffer)

Reagent	Final concentration	Amount
Normal Donkey Serum (NDS)	5%	2.5 mL
Bovine Serum Albumin (BSA, crystallized)	0.2%	100 mg
Sodium azide (5% in PBS)	0.05%	500 μL
Triton-X 100	0.3%	150 μL
Phosphate Buffered Saline (PBS, 1×)	1× (approx.)	47.35 mL
**Total**	**N/A**	**50 mL**

Keep at 4°C for up to 6 months or store aliquots at −20°C indefinitely.

**CRITICAL:** Always operate in a chemical fume hood and wear gloves to minimize the possible risks of working with concentrated sodium azide, which is a toxic substance that changes to a gaseous state after mixing with water.

**Table:** 4% PFA

Reagent	Final concentration	Amount
Paraformaldehyde (powder)	4%	20 g
Phosphate Buffered Saline (PBS, 2×, 4°C)	1×	250 mL
NaOH (10 M)	400 μM	20 μL
MQ water	N/A	250 mL
**Total**	**4%**	**500 mL**

Keep at 4°C for up to 1 week or at −20°C for up to 1 year.

**CRITICAL:** PFA is a moderately toxic substance and a carcinogen. Always operate in a chemical fume hood and wear gloves to minimize the possible risks of working with PFA.

***Alternatives:*** A ready-made 4% PFA in PBS is also commercially available from Histolab Products AB with the catalog number HL96753.1030.

**Table:** Mowiol mounting medium

Reagent	Final concentration	Amount
Mowiol 4–88 (pellets)	10%	2.4 g
Glycerol	20.8%	5 mL
MQ water	N/A	6 mL (add up to 24 mL)
Tris (0.2 M, pH 8.5)	0.1 M	12 mL
**Total**	**N/A**	**24 mL**

Keep at 20°C–25°C for active use or at −20°C indefinitely.

***Alternatives:*** There are commercially available alternatives for Mowiol mounting medium (e.g. Fluoromount Aqueous Mounting Medium by Sigma-Aldrich with the catalogue number F4680).

## Step-by-step method details

### PFA perfusion

**Timing: 10–20 min**

The purpose of PFA perfusion is to clear the blood from the vasculature while preserving the vessels to achieve the most optimal staining result.

***Note:*** For the protocol described here, we use adult mice of 20 weeks of age. Mice that are 6 weeks of age and older can be used, as there is no upper age limit in this protocol. However, for mice younger than 6 weeks of age, the perfusion method needs to be adjusted, which will not be discussed in this protocol.

1. Anesthetize the mouse.
  a. Administer a lethal dose of Ketaminol and Rompun anesthesia (300 mg/kg ketamine + 30 mg/kg xylazine) via intraperitoneal injection. Inject in the center of the lower quadra of the abdomen at a 45° angle using a syringe with a 30G needle.
  b. Wait until the mouse is completely anesthetized, i.e., has no responses to tail nor to toe pinches (approximately 3–5 min).

**CRITICAL:** From this step onward, operate in a chemical fume hood to minimize the possible risks of working with PFA, which is a moderately toxic substance and a carcinogen.

2. Insert the intake end of the peristaltic pump tubing into a container with ice-cold 2% PFA and flush the tubing and needle with 2% PFA to expel any air bubbles in the circuit.
3. Place the mouse on its back and pin its four paws to a Styrofoam board to secure the mouse during perfusion.
4. Spray 70% ethanol on the fur in the abdominal area to prevent stray fur from sticking to the internal organs.
5. Grip the chest skin using blunt forceps and make an incision along the skin using scissors to expose the peritoneum, then, expose the xiphoid, i.e., the piece of arrowhead-shaped white bone using scissors.
6. Grip the xiphoid with forceps and make lateral incisions on the peritoneum beneath the ribcage using scissors to expose the diaphragm and the liver.
7. Cut the diaphragm along the entire length of the ribcage using scissors.
8. Cut the ribcage on both sides up to the collarbone using scissors. Lift and pin the freed sternum with the detached ribcage to the Styrofoam board to expose the heart and the lungs.
9. Carefully remove the pericardial sac or other tissues covering the heart using blunt forceps to provide a clear view of the heart.
10. Insert the infusion needle attached to the peristaltic pump into the left ventricle at an angle approximately parallel to the midline of the heart.

**CRITICAL:** Insert the infusion needle about 5 mm into the left ventricle to avoid penetration into the left atrium or the right ventricle.

11. Make a small incision to the right atrium using fine scissors to allow the blood to exit from the circulation.
12. Start the perfusion immediately using a peristaltic pump and perfuse the mouse with 20 mL of ice-cold 2% PFA over 2 min. For the peristaltic pump used in this protocol, the speed is set to 41.1 rpm for this purpose. See troubleshooting: Problem 1, Problem 2.

***Note:*** The peristaltic pump enables better control of the perfusion speed, but manual perfusion with a syringe filled with 20 mL of ice-cold 2% PFA can also be used if a peristaltic pump is not available. In this case, adjust manual perfusion to 10 mL/min.

***Note:*** Successful perfusion can be determined by a color change in the liver and in the liquid flowing out of the right atrium. As PFA flushes out the blood, the color of the liquid flowing out turns from red to clear, and the liver gradually turns from red to beige.

13. Stop the perfusion and remove the infusion needle. Remove excess PFA/blood with paper towels and discard the PFA waste according to institutional rules and toxic waste disposal regulations.

### Collecting and cleaning the tissue sample

**Timing: 10–30 min**

The purpose of this step is to locate the correct area for collecting the tissue sample and clean the lumen of the intestinal sample from mucus and food remains to achieve the most optimal staining result.

**CRITICAL:** Operate in a chemical fume hood to minimize the possible risks of working with PFA.

14. Expose the abdominal organs by cutting the peritoneal wall from the xiphoid down to the symphysis pubis using scissors.
15. Carefully move the liver lobes aside using blunt forceps to provide a clear view of the stomach.
16. Grip the stomach with blunt forceps and cut the esophagus and any connective tissues using fine scissors, leaving only the duodenum and the rest of the small intestine attached to the stomach.
17. Unravel the small intestine from the rest of the gastrointestinal tract by gently pulling the stomach using blunt forceps and by carefully cutting any mesenteric connective and/or fat tissue using fine scissors.
  a. Unravel a minimum of 8 cm of the small intestine starting from the stomach and transfer the collected part to a measurement plate.

**Figure 1:**
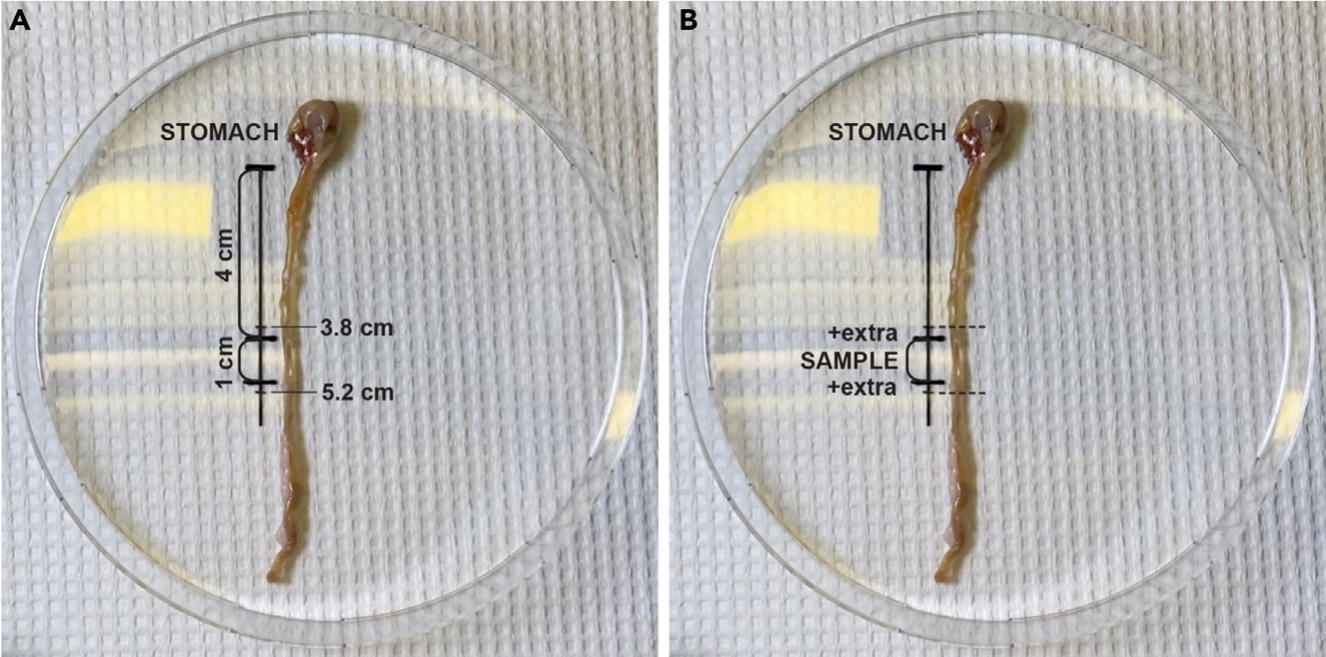
Obtaining the tissue sample (A) Determining the correct region for the sample using a measurement plate. (B) Cutting the tissue sample with extra tissue at both ends.

***Note:*** Transferring the tissue sample onto tissue paper can help to keep the sample in place while flushing.

***Note:*** Using a measurement plate ensures reproducible acquisition of the sample from the same region of the small intestine. For this protocol, we use a 1 cm long piece of the small intestine that is located between the 4 and 5 cm marks from the beginning of the duodenum (Figure 1).

18. Place the beginning of the duodenum to the 0 cm mark on the measurement plate and isolate the tissue sample by making incisions at marks 3.8 cm and 5.2 cm (Figure 1). See troubleshooting: Problem 3.

***Note:*** Leaving an additional 2 mm to both ends provides extra area for handling the tissue during the preparation. These extra ends are removed at a later stage.

**CRITICAL:** When handling the tissue sample, grip the sample from the end(s) of the intestinal tube and avoid gripping the sample from the center to keep the villi and vasculature intact.

19. Fill a syringe with 10 mL of ice-cold 4% PFA, attach a plastic gavage needle to the syringe, and remove any air.

**CRITICAL:** It is essential to use a flexible gavage needle to avoid damaging the intestinal wall and the fragile villi inside the intestinal tube during the cleaning procedure.

20. Grip one end of the tissue sample using blunt forceps and carefully insert the gavage needle into the lumen of the intestinal tube. Clean the lumen from mucus and possible food remains by flushing the lumen with 5 mL of ice-cold 4% PFA while moving the gavage needle back and forth within the lumen (Methods Video S1). See troubleshooting: Problem 1, Problem 2, Problem 4, Problem 5.

21. Grip the other end of the tissue sample using blunt forceps and repeat the flushing as in step 20 (Methods Video S1).
22. Place the cleaned tissue sample in ice-cold 4% PFA until dissection. See troubleshooting: Problem 5.

**Pause Point:** The cleaned tissue sample can be stored in ice-cold 4% PFA for up to 1 h before dissection.

Methods Video S1. Cleaning the sample, related to steps 20–21

### Dissecting, pinning, and fixing the sample

**Timing: 1 day**

The purpose of this step is to prepare the sample for whole-mount immunofluorescence staining.

***Note:*** We recommend performing dissecting and pinning of the sample using a dissection microscope to enable a better view of the sample.

**CRITICAL:** Handle the fine forceps and spring scissors carefully to avoid damaging the fine tips that bend and break easily. Thus, avoid touching hard surfaces with the tools’ tips, do not drop the tools, and always cover the tools’ tips accordingly after use.

23. Transfer the tissue sample to a silicone-coated plate containing ice-cold PBS.
24. Remove any remains of the mesentery and/or fat attached to the sample using fine forceps and spring scissors.
25. Cut off the extra 2 mm from both ends of the intestinal tube to provide straight and even edges for later stages (Methods Video S2).
26. Cut the intestinal tube open longitudinally using spring scissors (Methods Video S2).

***Note:*** It is best to have even and straight cuts on all four sides of the tissue sample. This makes cutting the intestine strips easier in the upcoming phases.

27. Pin down the tissue sample flat onto a silicone-coated plate so that the villi are facing upwards (Methods Video S2).
  a. Pin the four corners of the tissue sample onto the silicone-coated plate with insect pins using blunt forceps. Gently stretch the tissue piece while pinning to ensure that the tissue piece is pinned down as flat and straight as possible.

**CRITICAL:** Handle the insect pins with blunt forceps, fine forceps can break easily while handling the pins.

28. Carefully remove possible dirt and/or mucus from the villi surface using curved forceps.
29. Working in a fume hood, remove PBS and add ice-cold 4% PFA to the plate. See troubleshooting: Problem 5, Problem 6.

**CRITICAL:** Use enough volume of solutions to completely cover the tissue sample without overflow.

30. Place the lid on the plate and seal the plate using parafilm to prevent the liquid from leaking. See troubleshooting: Problem 6.
31. Fix the sample in 4% PFA at 4°C with rotation for 16–24 h. See troubleshooting: Problem 1, Problem 2, Problem 5, Problem 6, Problem 7.

**Pause Point:** The sample should be fixed for 16–24 h.

32. Working in a fume hood, remove 4% PFA after the fixation and wash the sample with PBS at 20°C–25°C with rotation for 4 × 30 min.

**CRITICAL:** PBS used for washing away the PFA should be discarded as PFA waste from this point onward.

Methods Video S2. Dissecting and pinning the sample, related to steps 25–27

### Blocking and whole-mount immunofluorescence staining

**Timing: 3 days**

The main purpose of this step is to stain the small intestine sample using an antibody specific for podocalyxin antigen to visualize blood vessels and an antibody against lymphatic vessel endothelial hyaluronan receptor 1 (LYVE1) antigen to visualize lymphatic vessels. Podocalyxin is expressed on the luminal surface of the vasculature and is a useful marker for detecting blood and lymphatic vessels in various tissues.^2^^,^^3^^,^^4^ LYVE1 is a more specific marker for mainly initial (capillary) lymphatic vessels. This protocol has also been used for other stainings using antibodies against vascular endothelial growth factor receptors 2 and 3 (KDR, FLT4), prospero homeobox protein 1 (PROX1), ETS-related gene (ERG), and vascular endothelial (VE)-cadherin (CDH5) in the adult murine small intestines.

33. For permeabilization, remove PBS and wash the sample with 0.3% PBST at 20°C–25°C with rotation for 4 × 30 min. See troubleshooting: Problem 1.
34. Remove 0.3% PBST and block the sample in DIM at 20°C–25°C with rotation for 1 h.

**CRITICAL:** Blocking time should not be shorter than 1 h to reduce unspecific staining.

35. For immunodetection of the antigens, remove DIM and incubate the sample in primary antibodies (goat anti-podocalyxin, 1:400 and rabbit anti-LYVE1, 1:600) diluted in DIM at 4°C with rotation for 2 days. See troubleshooting: Problem 1, Problem 2, Problem 6.

**Pause Point:** The sample should be incubated in primary antibody mix for 2 days.

36. Remove primary antibody mix and wash the sample with 0.3% PBST at 20°C–25°C with rotation for approximately 6 h, changing the solution at least 5 times during this time.
37. For the fluorescence detection of the primary antibodies, remove 0.3% PBST and incubate the sample in secondary antibodies (donkey anti-goat Alexa Fluor™ Plus 488, 1:1000, and donkey anti-rabbit Alexa Fluor™ Plus 594, 1:1000) diluted in PBS at 4°C for 16–24 h with rotation and protected from light. See troubleshooting: Problem 1, Problem 2, Problem 6, Problem 7.

**Pause point:** The sample should be incubated in secondary antibody mix for 16–24 h.

**CRITICAL:** From this step onward, protect the sample from light during longer incubations by, for example, covering the plate with aluminum foil.

***Note:*** In our experience, the most optimal staining results are achieved using secondary antibodies with Alexa-488 or Alexa-594 conjugates, while staining with Alexa-647-conjugated secondary antibodies may result in poorer staining intensity.

***Note:*** Centrifuge the secondary antibodies at a speed of ≥17,000 g for 5 min at 20°C–25°C to pellet any aggregates and use only from the supernatant to avoid having speckled staining while imaging.

38. Remove secondary antibody mix and wash the sample with PBS at 20°C–25°C with rotation for approximately 6 h, changing the solution at least 5 times. See troubleshooting: Problem 7.
39. Working in a fume hood, remove PBS and post-fix the sample with 1% PFA at 20°C–25°C with rotation for 10 min.
40. Working in a fume hood, remove PFA and wash the sample with PBS at 20°C–25°C with rotation for 2 × 15 min.

**Pause point:** The post-fixed tissue sample can be stored in PBS at 4°C for up to 7 days before mounting.

### Mounting

**Timing: 5–30 min**

The primary purpose of this step is to mount the stained small intestine samples to enable the visualization of the vasculature using a microscope. Thus, it is crucial to mount thinner intestine strips rather than the whole tissue sample to allow for clear visualization of the entire villus vasculature.

41. Label a microscope slide with necessary information about the sample (e.g., stainings, experiment information, sample identification number, etc.).
42. Under a dissection microscope, remove any dirt attached to the villi using curved forceps and reposition the pins using blunt forceps, if needed.
43. Cut 1-2 villi thick strips from all four edges of the sample using spring scissors (Methods Video S3). See troubleshooting: Problem 1.

**CRITICAL:** The intestine strips should be 1–2 villi thick because strips that are ≥3 villi thick are challenging to mount as they curl more easily and the submucosa can fold on top to cover the villi, which disturbs imaging of the entire villi vasculature.

44. Place a small droplet of Mowiol mounting medium on one side of a microscope slide (Methods Video S3). See troubleshooting: Problem 1.

***Note:*** It is recommended to avoid using Mowiol mounting medium when imaging samples containing fluorescent proteins, such as those obtained from green fluorescent protein (GFP) or red fluorescent protein (RFP) reporter mouse lines as Mowiol may lead to decreased fluorescence intensity of these proteins. In such cases, it is advised to use an alternative mounting medium.

**Figure 2:**
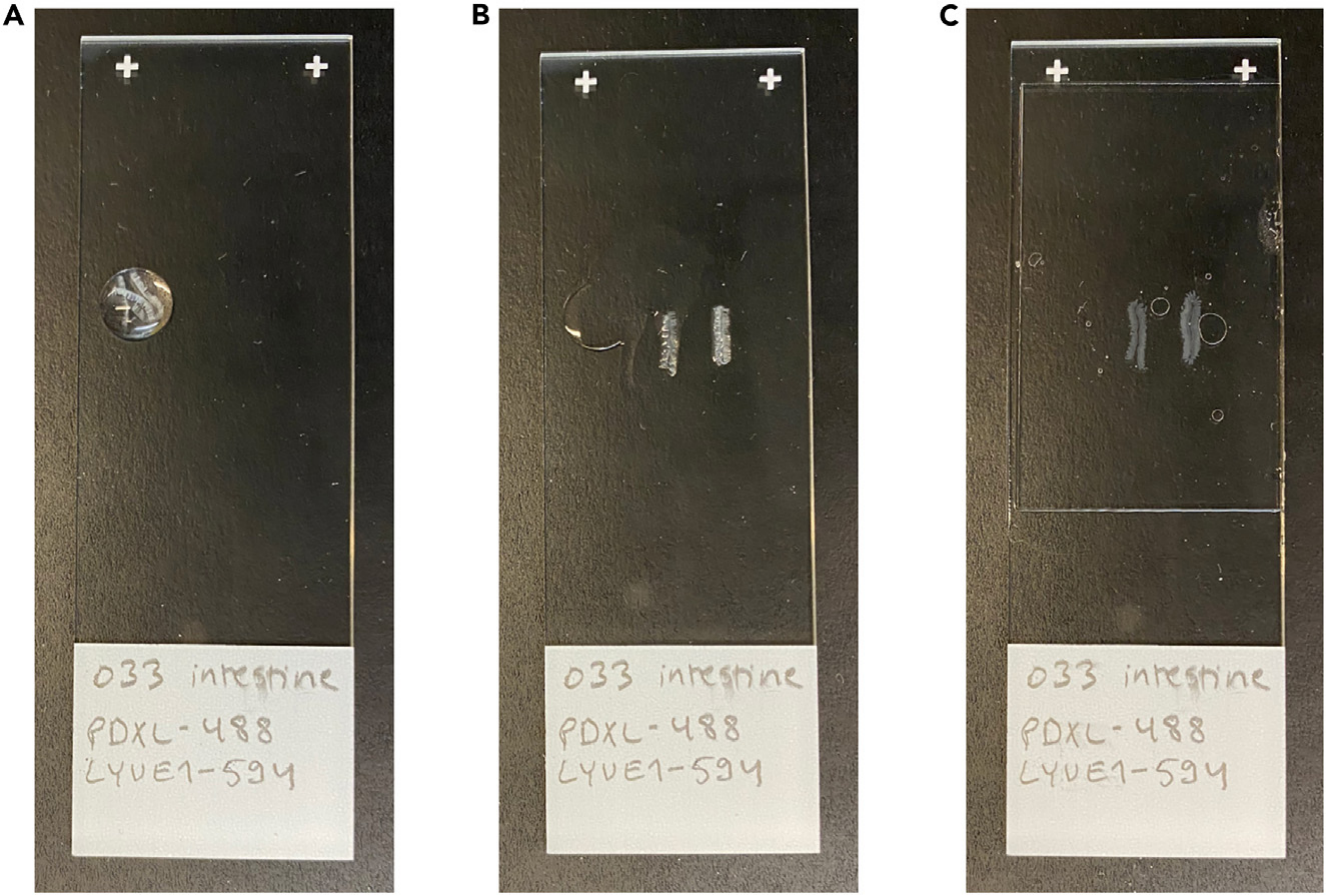
Mounting the intestine strips (A) Placing the intestine strips into mounting medium droplet on the microscope slide. (B) Positioning the intestine strips on the microscope slide. (C) Mounted intestine strips.
46. Position the strips to the center of the microscope slide using fine forceps so that two strips are positioned back-to-back (Methods Video S3, Figure 2B).
  a. Grab a strip from one end and carefully pull it from the mounting medium droplet towards the center of the microscope slide. Ensure that the strip is aligned parallel to the longer edge of the microscope slide as you position it.
  b. Ensure that all the villi of a single strip face the same direction.
  c. Grab and pull another strip and position it adjacent to the first strip so that the submucosal side of the strips are in contact while ensuring that the villi face outwards.

***Note:*** While the back-to-back orientation of the strips helps to save space and keeps them straight during mounting, the strips can also be positioned individually if desired.

***Note:*** It is possible to mount multiple samples on the same slide using this method. However, it is important to work quickly to prevent the mounting medium droplets from drying, and to ensure there is enough space between the samples. Additionally, a large enough cover slip should be used to cover all the samples adequately.

45. Transfer the strips into the mounting medium droplet on the microscope slide using fine forceps (Methods Video S3, Figure 2A).

47. Place a cover glass on top of the sample(s) (Figure 2C).
48. Fill up the remaining space between the microscope slide and the cover slip with Mowiol mounting medium using a 200 μL pipette. Avoid air bubbles.
49. Seal all four edges of the cover glass with nail polish and let dry.
50. Store the mounted sample horizontally on a flat surface at 4°C and protect it from light until imaging.

**Pause point:** The mounted sample can be stored at 4°C protected from light for up to 4 weeks if it is well-sealed. However, it is recommended to image the samples within 3 days after mounting for optimal results.

***Note:*** Storing the whole-mount samples vertically may cause the mounting medium to leak and lead to the drying of the samples. It is recommended to store the samples horizontally to prevent this from happening.

51. Image the samples. See troubleshooting: Problem 2.

***Note:*** Our laboratory utilizes a ZEISS LSM 780 confocal microscope with ZEN 2012 software, along with either a 10× Plan-Apochromat objective with a numerical aperture (NA) of 0.45 or a 20× Plan-Apochromat objective with a NA of 0.80, both of which are air objectives. We typically image small intestine samples that have been stained with Alexa-488, Alexa-594, or Alexa-647-conjugated secondary antibodies. However, depending on the experimental design, other magnifications, objectives, and fluorophores may be utilized to achieve desired imaging outcomes.

Methods Video S3. Cutting and mounting the intestine strips, related to steps 43–46

## Expected outcomes

**Figure 3:**
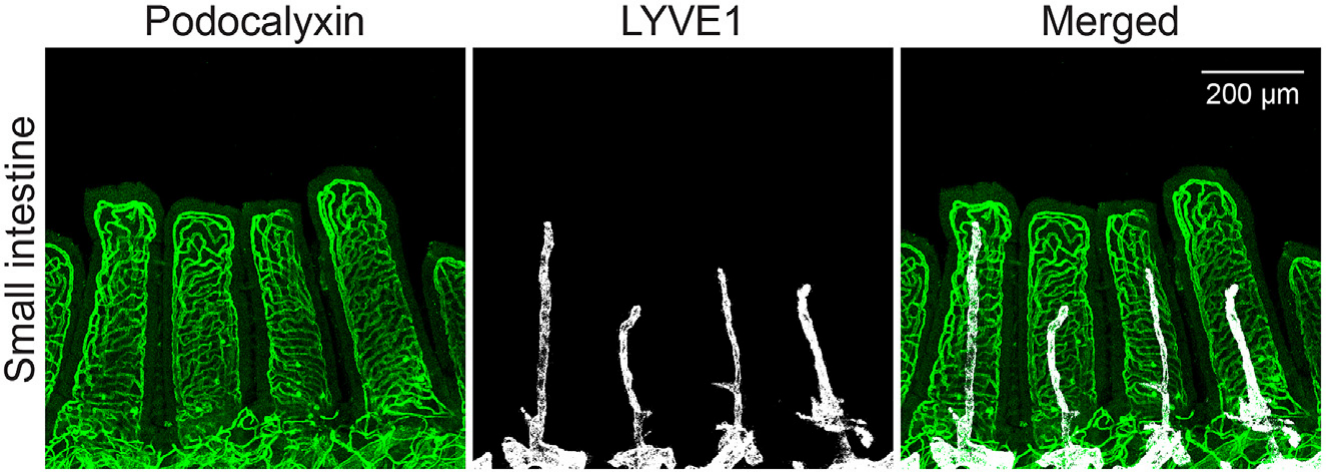
Representative confocal micrographs of whole-mounted small intestine stained for podocalyxin (green, all vessels) and LYVE1 (white, lymphatic vessels) Scale bar: 200 μm.

This protocol describes a fast and simple method to visualize the blood and lymphatic vasculature in the murine small intestine, specifically in the duodenum. However, it is important to note that other excellent methods papers describe whole-mount staining and visualization of the murine intestine, such as those by Bernier-Latmani and Petrova (2016) and Hatch and Mukouyama (2015).^5^^,^^6^ In this protocol, the anti-podocalyxin antibody stains both blood and lymphatic vessels, while the anti-LYVE1 antibody is used to specifically stain lymphatic vessels in the submucosa and villi of the small intestine. The resulting staining should allow the visualization of typical vascular structures, as shown in Figure 3. Of note, using an anti-podocalyxin antibody results in somewhat dimmer staining on the lymphatic endothelial cells of the lacteals compared to blood vessels of the villi (Figure 3). If LYVE1-positive single cells are observed along the blood vessels, these are likely tissue-resident macrophages that express the LYVE1 antigen.

Each villus has a central core composed of one artery and one vein, a strand of muscle, a centrally located lymphatic capillary (lacteal), and connective tissue that supports the villus structure. The blood vessels are thought to transport proteins, short-chain fatty acids, and carbohydrates absorbed and released by the enterocytes of the villi, while the lymphatic capillary transports longer-chain fatty acids and chylomicrons. Genetic manipulations or different diseases may affect the density, width, and branching properties of the blood vessels and the length and width of the lymphatic vessels.

## Limitations

The current protocol is effective for obtaining a general understanding of the vascular morphology in the small intestine. However, a different preparation and imaging approach would be required to acquire a three-dimensional perspective of the villi vasculature.

## Troubleshooting

### Problem 1

The staining is dim and/or uneven (Steps 12, 20, 31, 33, 35, 37, 43, and 44).

### Potential solution

Faint staining can result from several factors. To avoid incomplete fixation, ensure that the perfusion fixation is successful (a change in color of the liver tissue from dark red to beige) (Step 12) and make sure the tissue is fixed in 4% PFA for at least 16 h (Step 31). This is a critical step, and if the tissue is incompletely fixed, the experiment needs to be repeated. The intestinal lumen should also be properly cleaned as instructed using ice-cold 4% PFA to remove mucus and digestive material, including enzymes and bacteria, which can degrade the tissue and hinder antibody penetration and antibody-antigen interaction (Step 20). In addition, the permeabilization should be at least 2 h in total (Step 33) to allow antibody penetration and antigen-antibody interaction. To enhance staining efficiency, it is important to incubate primary antibodies for at least 2 days (Step 35) and secondary antibodies for 16–24 h (Step 37). If using different antibodies than those specified in this protocol, adjusting the primary antibody concentration may be necessary. We recommend using secondary antibodies with Alexa-488 or Alexa-594 conjugates, as our experiments have shown poor staining results with Alexa-647-conjugated secondary antibodies. To obtain the best staining results, it is advisable to cut intestine strips from the edges of the tissue sample, as the tissue edges typically exhibit the strongest staining (Step 43). Additionally, Mowiol mounting medium should not be used with reporter mouse lines, as it can reduce the signals of fluorophores such as GFP, RFP, or tdTomato (Step 44). For these fluorophores, we advise VECTASHIELD® Antifade mounting medium from Vector Laboratories with the catalog number H-1000-10 to be used instead.

### Problem 2

There is no staining (Steps 12, 20, 31, 35, 37, and 51).

### Potential solution

Inadequate staining can result from several factors. To avoid incomplete fixation, ensure that the perfusion fixation is successful (a change in color of the liver tissue from dark red to beige) (Step 12) and make sure the tissue is fixed in 4% PFA for at least 16 h (Step 31). This is a critical step, and if the tissue is incompletely fixed, the experiment needs to be repeated. The intestinal lumen should also be properly cleaned as instructed using ice-cold 4% PFA to remove mucus and digestive material, including enzymes and bacteria, which can degrade the tissue and hinder antibody penetration and antibody-antigen interaction (Step 20). If the tissue was fixed well, but an antibody was forgotten, one can perform the staining steps starting from the step with the potentially missing antibody (Steps 35 and 37). We recommend repeating all co-stainings in this case, as further washes might reduce the staining intensity. If a non-functional antibody batch was used, the staining can be repeated on the fixed tissue using a new batch of antibody that is tested for its functionality, by repeating all co-stainings (Steps 35 and 37). Additionally, problems with the imaging set up can hinder visualization of the staining (Step 51); thus, it is also important to confirm that the microscope used for imaging is functioning properly, and that the shutter position is correct. Ensure that the objective is clean and the view through it is not obstructed, the lasers required for the imaging are online and functional and the camera connection to the software is uninterrupted. Alternatively, check the stainings on a different microscope to see if the problem arises from the sample or the microscope.

### Problem 3

The villus morphology does not correspond to the example photos (Step 18).

### Potential solution

The morphology of villi (including the thickness and length) varies depending on the region of the gastrointestinal (GI) tract and the age of the mouse. In general, villi thickness increases, and villi length decreases towards the lower GI tract. In this protocol, we obtain tissue samples from the duodenum of a 20-week-old mouse. It is important to consider the age of the mouse when selecting the region of the GI tract from which to obtain tissue samples, as obtaining samples from a younger or older mouse may result in a different region of the GI tract to be collected. To ensure consistency among mice, a measurement plate can be used to obtain tissue samples from similar regions. It is also important to note that genetic, therapeutic, or chemical manipulations may alter villi morphology or the structures within.

### Problem 4

The gavage needle could not be inserted into the intestinal tube (Step 20).

### Potential solution

The gavage needle’s round tip may be too large to be inserted into the intestinal tubes, especially in younger or smaller mice. If this is the case, cutting off the tip can help insert the needle into the lumen more easily, but it may also result in a sharper tip that could damage the villi. Inserting the gavage needle can also be challenging if the tissue sample is dry. To prevent the sample from drying out, it is recommended to dip the sample in 1% PFA or PBS.

### Problem 5

The villi are damaged and/or villus coverage is uneven or sparse (Steps 20, 22, 29, and 31).

### Potential solution

Villi are fragile structures that can be easily damaged. Proper flushing of the intestinal tube with ice-cold 4% PFA is crucial to clean the lumen of digestive material, including enzymes and bacteria, which can degrade tissue and damage villi (Step 20). To prevent further damage, the cleaned sample should be kept in ice-cold 4% PFA (Step 22), and PFA fixation should begin as soon as possible after pinning down the sample (Steps 29 and 31). Additionally, it is important to note that the mouse may have pre-existing villi damage or sparse villi coverage due to disease or genetic modifications.

### Problem 6

The sample pinned to the silicone-coated plate has dried (Steps 29–31, 35, and 37).

### Potential solution

It is important to ensure that the lid of the silicone-coated plate is securely sealed using Parafilm to prevent leakage during the staining process. Additionally, overfilling the plate can also lead to liquid spilling out when placed on a rotator. To avoid this, the optimal liquid volume should cover the tissue sample entirely without exceeding the capacity of the plate.

### Problem 7

The stained sample shows high fluorescence background during imaging (Steps 31, 37, and 38).

### Potential solution

High background staining can result from several factors. To minimize high background staining, it is important to avoid over-fixation (> 24 h) (Step 31), which can result in elevated background staining in the detection channel of Alexa-488-conjugated secondary antibodies. In addition, it is important to avoid using excessively high concentrations of secondary antibodies and to ensure that the recommended incubation times for secondary antibodies are not exceeded, as both can contribute to increased background staining in all channels (Step 37). Finally, adequate washing of the samples after the secondary antibody incubation step is crucial for achieving an optimal signal-to-background ratio (Step 38).

## Resource availability

### Lead contact

Further information and requests for resources and reagents should be directed to and will be fulfilled by the lead contact, Sinem Karaman (sinem.karaman@helsinki.fi).

### Materials availability

This study did not generate new unique reagents.

## Data Availability

This study did not generate datasets or code.
